# Breast Cancer Sentinel Lymph Node Detection Rate: First Large Scale Multi-Centric Data for Technetium Phytate

**DOI:** 10.34172/aim.2023.91

**Published:** 2023-11-01

**Authors:** Ramesh Omranipour, Mehrshad Abassi, Newsha Nazarian, Bardia Gholami, Samareh Heydari, Bita Eslami, Alireza Abdollahi, Sadaf Alipour

**Affiliations:** ^1^Breast Disease Research Center, Cancer Institute, Tehran University of Medical Sciences, Tehran, Iran; ^2^Department of Cancer Surgery, Cancer Institute, Tehran University of Medical Sciences, Tehran, Iran; ^3^Department of Nuclear Medicine, Vali-Asr Hospital, Imam Khomeini Hospital Complex, Tehran University of Medical Sciences, Tehran, Iran; ^4^Faculty of Medicine, Tehran Medical Branch, Islamic Azad University; ^5^Faculty of Medicine, Shahid Beheshti University of Medical Sciences, Tehran, Iran; ^6^Department of Pathology, Imam Khomeini Hospital Complex, Tehran University of Medical Sciences, Tehran, Iran; ^7^Department of Surgery, Arash Women’s Hospital, Tehran University of Medical Sciences, Tehran, Iran

**Keywords:** Axillary staging, Breast cancer, Radioisotope scan, Sentinel node, ^99m^Technetium phytate

## Abstract

**Background:**

Sentinel lymph node biopsy (SLNB) with injection of radiopharmaceuticals is now the standard of care for staging the axilla in patients with breast cancer. Sulfur or antimony colloids labeled with ^99m^Technetium (Tc) are used globally for the procedure, with a detection rate of 94%. However, in Iran, Tc phytate has been used because it is more easily producible in the country. The detection rate with Tc phytate has not been well determined in large-scale studies.

**Objective:**

We performed this study to report the detection rate of SLNB with Tc phytate, its advantages and disadvantages using large multicentric data.

**Methods:**

This is a retrospective cross-sectional multicenter study. Participants were breast cancer patients without previous history of axillary surgery, who underwent sentinel node biopsy using Tc phytate on the morning of surgery or the day before. The detection rate was calculated as the number of patients with histologically positive sentinel nodes to all patients with histologically positive lymph nodes; we compared those injected on the day of surgery and those injected on the day before.

**Results:**

Overall, 2663 women aged 50.2±11.6 years were included. The detection rate was 91.8% (806 out of 878). The false negative rate was 8.2% overall, and statistically similar for injections on the day or the day before surgery (2.9 vs 2.1; *P*=0.32).

**Conclusion:**

Tc phytate has a good detection rate for breast radio-guided SLNB with similar result for injections on the surgery day or the day before it.

## Introduction

 Sentinel lymph node biopsy (SLNB) has become the standard of care for staging the axilla in patients with breast cancer.^1,2^ The procedure reduces the need for axillary lymph node dissection and its consequent complications.^3,4^ SLNB could be performed with the visual guide by injections of dye or an auditory guide of radiopharmaceuticals. Globally, sulfur colloid or antimony colloids labeled with ^99m^Technetium (Tc) have been used extensively for SLNB with documented high detection rates of 94% (93-95), usually better but overlapping the detection rate of a visual guide by dye injection.^5,6^ The axillary recurrence is very low (i.e. 0.3%) in patients with negative primary SLNB.^5^ For more than a decade, Tc phytate, a radiopharmaceutical different from tracers used globally, has been employed in Iran. Tc phytate is different from other colloid radiopharmaceuticals used for SLNB essentially in terms of the colloid size; actually, Tc phytate is not a colloid, and the biological reactions after injections with calcium provide a final product which is colloid. The detection rate of this radiopharmaceutical has not been determined in large-scale multicentric studies up to now; however, breast surgeons in Iran have been using this agent with good practical results. The washout of the tracer had been a concern so that a decade ago, the injections the-day-before surgery were prohibited until good accuracy of the early injections were confirmed for Tc sulfur colloid and antimony. Similar concerns are remarkable for Tc phytate mainly because the particle size is estimated to be smaller and the washout may occur further. The current study reports the largest multicentric data of SLNB with Tc phytate to date. In this paper, the detection rate of SLNB by Tc phytate was calculated and the advantage and disadvantages of the use of this radiopharmaceutical are discussed.

## Materials and Methods

###  Study Design

 This is a retrospective cross-sectional multicenter study held in three referral centers for breast cancer (Tehran, Iran). The study received the Ethics Approval Code from the Ethics Committee of Tehran University of Medical Sciences (IR.TUMS.SINAHOSPITAL.REC.1400.048) and was approved by the Research Deputy of the University (Approval Code: 1400-1-101-52637). Data were extracted from the records of patients who were hospitalized for breast cancer surgery. Patients had given their written informed consent for the use of their data in research at the time of hospitalization.

###  Setting

 Radioisotope injections and optional scans were performed by nuclear medicine specialists at the nuclear medicine units (NMUs). At one of the study centers, the NMU was located in the hospital but in the two other study centers, the NMUs were located elsewhere. Tc phytate (Pars Isotope Co, Tehran, Iran) was prepared in nuclear medicine centers and injected via 2 to 4 peri-areolar subdermal injections on the morning of surgery or the day before surgery such that between 12 to 37 mBq Tc phytate was present at the time of surgery. Three oncologic breast surgery experts who were professors at Tehran University of Medical Sciences carried out the sentinel node biopsy and further dissection as needed. The following gamma probes were used for SLNB: Europrobe 3.2 (Eurorad, Eckbolsheim, France,) and Sergeoguide (Parto Negar Persia, Tehran, Iran).

 At first, the names and file numbers of patients who had undergone sentinel node biopsies from 2007 to 2021 were extracted from the pathology databases of the study centers. In these centers, the first sentinel node biopsy procedures started practically and officially in 2007, after passing the learning curve. Then, the medical records of those eligible for this study were reviewed and data were extracted from the medical notes and histology results of the hospital files. Data extraction was carried out by three medical students under the supervision of the principal investigator.

 The sample size was not calculated before the study; considering a purposive sampling method, based on our general view about the incoming patients, we expected to include at least 2200 cases. During the project, we identified the records of 2771 patients.

###  Eligibility Criteria

 Participants who were eligible for inclusion were female patients with breast cancer at any age who had undergone sentinel node biopsy by use of a radioisotope.

 Exclusion criteria consisted of recurrent breast cancer, previous axillary surgery, use of blue dye alone for sentinel node biopsy, and incomplete records about the tumor or the biopsied and dissected lymph nodes.

###  Variables and Outcomes 

 Variables extracted from the records consisted of age, tumor characteristics, number of free or involved sentinel and non-sentinel nodes, number of free and involved nodes in case of axillary dissection, and the protocol of radioisotope injection. The latter consisted either of the two-day protocol, which comprised injection of the tracer within 24 hours on the day before surgery; or the one-day protocol, which included injection on the day of the operation.

 Sentinel nodes (SN) consisted of those detected by the radioisotope tracer and hand-held gamma counter; non-sentinel nodes (NSN) were the nodes that had not absorbed any tracer but were excised by the surgeon during SN biopsy (SNB) due to their larger size or firmer consistency. Dissected nodes (DN) were those that were excised during lymph node dissection via the axilla in cases where the SN or NSN was positive.

 The number of excised nodes, free nodes, and involved nodes among the SN, NSN and DN were recorded according to the pathology reports. Also, the tumor characteristics including tumor histologic type, grade, and lymphovascular invasion were collected. Tumor type was defined based on the 5^th^ edition of the WHO classification of tumors of the breast.^7^

 The primary outcome was the detection rate, defined as the number of positive SNs divided by the sum of the patients with positive lymph node involvement in SNs, NSN, or DN; a false negative SN was defined as a negative SN in the presence of positive NSNs or positive DNs. The secondary outcome was the difference in the detection rate between the one-day or two-day protocols.

###  Bias

 The accuracy of sentinel node biopsy might not be dependent only on the type of tracer, and the surgeons’ experience may bias the results. Also, as the study encompasses 14 years of practice, the experience of surgeons who had performed the surgery has surely evolved throughout time; this could lead to a discrepancy in results obtained at different times; but the result is generalizable because the pooled detection of the long study period comprises different experience statuses of the surgeons.

###  Statistical Methods

 All analyses were performed using SPSS version 24 (IBM SPSS Statistics for Windows, version 24.0, 2016, NY: IBM Corp). Continuous variables were presented as mean ± standard deviation and categorical variables were presented as numbers and percentages. Median and interquartile ranges (IQR) were presented when appropriate. Comparison between the results of one-day and two-day isotope injection protocols was made using the chi-squared test. The necessary assumptions were assessed for the chi-squared test including no cell counts fewer than 1 patient and at most 20% of expected cells counts fewer than 5 cases; Fisher exact test was employed when necessary. A *P* value less than 0.05 was considered statistically significant.

## Results

###  Participants 

 Overall, data of 2771 patients who had undergone SLB were extracted from the files. A total of 108 cases were excluded due to previous axillary surgery or the use of blue dye for sentinel node biopsy. Finally, data from 2663 cases were analyzed.

###  Descriptive Data, Outcome Data and Main Results

 The patient and tumor characteristics are presented in Table 1. At least 1 SLN (median = 2; range: 1‒24; and IQR: 1‒3) was detected in 2659 patients; detection rate was calculated at 99.8% (95% CI: 99.7‒100.0%). Overall, 806 patients had at least one positive SN. In patients with negative SNs (n = 1857), 72 patients had either positive NSN or positive DN. The false negative rate was calculated at 8.2% (95% CI: 6.4‒10) and 2.7% (95% CI: 2.1‒3.3) for patients with LN metastases and all patients, respectively. The sensitivity was 91.8 (95%CI: 90‒93.6; 806 out of 878). False negative rates were statistically similar for one- and two-days protocols (2.9 vs 2.1 total 2.7%; Fisher’s exact test *P* = 0.32). The odds ratio for a false negative result in two-days vs. one-day protocol was 1.4 (95%CI: 0.8‒2.0). The number of patients with excised SN, NSN, and DN as well as the rate of positive LN detection and false negative status rate of SLNs are presented in Table 2.

**Table 1:** Characteristics of the Patient’s Tumors and the Injection Protocol

**Patient or Tumor Variable **		**Mean±SD or Number (%)**
Age (y)		50.2 ± 11.6
Tumor size (mm)		23.5 ± 13.3
Tumor side	Left	1360 (51)
	Right	1289 (48.4)
	missing	14 (0.5)
	Total	2663 (100)
Tumor type	DCIS	215 (8.8)
	IDC	1935 (78.8)
	ILC	202 (8.2)
	LCIS	11 (0.4)
	Others	92 (3.7)
Tumor grade	1	319 (13.7)
	2	1422 (61.2)
	3	581 (25)
Isotope injection protocol	1-day	2046 (76.8)
	2-days	617 (23.2)

DCIS, Ductal carcinoma in-situ; IDC, invasive ductal carcinoma; ILC, Invasive lobular carcinoma; LCIS, Lobular carcinoma in situ; SD, standard deviation.

**Table 2:** Number and Involvement Status of Sentinel, Non-sentinel, and Dissected Lymph Nodes, and the Detection and False Negative Sentinel Lymph Node Rates

**Patient or Tumor Variable **		**Number (%)** **(N=2663)**
SN status	Negative	1853 (69.6)
	Positive	806 (30.3)
	Not detected	4 (0.2)
	Detected	2659 (99.8)
NSN status	Negative	794 (29.8)
	Positive	113 (4.2)
	Resected	907 (34.1)
	Non-existent	1756 (65.9)
DN status	Negative	976 (36.7)
	Positive	297 (11.2)
	Not dissected	1390 (52.2)
Result of SN biopsy	True negative	1785 (67.0)
	Positive	806 (30.3)
	False negative	72 (2.7)

SLN, Sentinel lymph node; NSN, Non- sentinel lymph node; DN, Dissected lymph nodes.

## Discussion

 For the first time in a multi-center large-scale study, a high detection rate of 99.8% has been documented for SLNB with Tc phytate. This figure is high and comparable with the results of other radioisotopes employed in other regions of the world. The best detection rate is achievable with dual techniques measured at 97% for breast cancer^8^ and 99% for advanced disease before neoadjuvant therapy.^9^ The false negative rate of phytate was 8.2% in the current study with the largest sample ever but a diverse population. This rate is comparable with previous reports of 7.1% (2 out of 28),^10^ 9.1%,^11^ 15.6% (7 out of 45),^12^ and 19.0% (4 out of 21)^13^ in smaller studied populations. There is no head-to-head comparison but the diagnostic accuracy of the Tc phytate in current large-scale study is equal or superior to the more globally used radioisotopes. In meta-analyses, the false negative rates of other radioisotopes are estimated at about 3%,^14^ 5%,^15^ 7.5%,^16^ 8%,^17^ 10%,^18^ and up to 12%^19^ which are quite comparable with the results of the current study concerning Tc phytate.

 Furthermore, we demonstrated that the detection rates of procedures with injections on the day of surgery or the day before surgery are the same and suspicions raised about the washout of the primary SLNs would not be valid for Tc phytate similar to other radioisotopes, which are globally more employed.

 Phytate has a remarkable advantage over sulfur colloid and antimony-based radiopharmaceuticals, which include its simple preparation procedure, no need to boil, and its shorter preparation time. Tc phytate is prepared with just a 2-minute shaking process at room temperature and becomes ready to inject after 20 minutes.^20^ The disadvantages of this radiopharmaceutical include the *in-vivo* colloid formation after injection which brings certain suspicions about the colloid size, and the possible washout of the tracer out of the primary SN due to possible instability.^21^ In Iran, phytate is used instead of the sulfur colloid and antimony (which are respectively used in the United States and Europe) because it is more easily producible inside the country, although Tc antimony is available, and has a cost reduction privilege over other tracers. The current study documented a detection rate similar to other radiopharmaceuticals and with similar on-the-day or on-the-day-before-surgery diagnostic accuracy.

 This study had some limitations; first, it suffers from the pitfalls of retrospective studies. The data were not collected for research purposes and were registered by the treating surgical team. Furthermore, the exact dose of radiopharmaceuticals was not recorded and could not be assessed. Lastly, the effect of experience and learning process on the results was not under control. However, considering that the data were collected during a long-time lapse of 14 years with surgeons becoming more experienced during the study period as well as the inclusion of patients with different injection methods in different nuclear medicine centers (i.e. variable exact site, depth, and dose of injection), we believe that the result is generalizable for different surgical and nuclear medicine settings.

## Conclusion

 In conclusion, the results indicate that Tc phytate is a reliable radiopharmaceutical for breast SLNB with robust results with injection either on the day or the day before surgery. Considering the easier preparation method, we suggest that Tc phytate may replace the more routinely used agents.
