# Correction: Breast Cancer Sentinel Lymph Node Detection Rate: First Large Scale Multi-Centric Data for Technetium Phytate

**DOI:** 10.34172/aim.33700

**Published:** 2025-01-01

**Authors:** Ramesh Omranipour, Mehrshad Abbasi, Newsha Nazarian, Bardia Gholami, Samareh Heydari, Bita Eslami, Alireza Abdollahi, Sadaf Alipour

**Affiliations:** ^1^Breast Disease Research Center, Cancer Institute, Tehran University of Medical Sciences, Tehran, Iran; ^2^Department of Cancer Surgery, Cancer Institute, Tehran University of Medical Sciences, Tehran, Iran; ^3^Department of Nuclear Medicine, Vali-Asr Hospital, Imam Khomeini Hospital Complex, Tehran University of Medical Sciences, Tehran, Iran; ^4^Faculty of Medicine, Tehran Medical Branch, Islamic Azad University, Tehran, Iran; ^5^Faculty of Medicine, Shahid Beheshti University of Medical Sciences, Tehran, Iran; ^6^Department of Pathology, Imam Khomeini Hospital Complex, Tehran University of Medical Sciences, Tehran, Iran; ^7^Department of Surgery, Arash Women’s Hospital, Tehran University of Medical Sciences, Tehran, Iran

 This notice corrects the article titled “Breast Cancer Sentinel Lymph Node Detection Rate: First Large-Scale Multi-Centric Data for Technetium Phytate,” published in 2023; 26(11): 618-622 (DOI: 10.34172/aim.2023.91). In the original version, the second author’s name was misspelled as “Mehrshad Abassi” in both the author list and the Authors’ Contribution. The correct spelling is “Mehrshad Abbasi.”

 This correction has been implemented in both the PDF and HTML versions of the article.

